# Biomaterial-based platforms for cancer stem cell enrichment and study

**DOI:** 10.20892/j.issn.2095-3941.2020.0420

**Published:** 2021-06-15

**Authors:** Chunhua Luo, Zhongjie Ding, Yun Tu, Jiao Tan, Qing Luo, Guanbin Song

**Affiliations:** 1 Key Laboratory of Biorheological Science and Technology, Ministry of Education, College of Bioengineering, Chongqing University, Chongqing 400044, China; 2 Institute of Pathology and Southwest Cancer Center, Southwest Hospital, Third Military Medical University (Army Medical University), Key Laboratory of Tumor Immunopathology, Ministry of Education of China, Chongqing 400038, China; 3 School of Pharmacy, Chongqing Medical and Pharmaceutical College, Chongqing 401331, China

**Keywords:** Cancer stem cell, biomaterial, CSC enrichment and study, three-dimensional culture platform

## Abstract

Cancer stem cells (CSCs) are a relatively rare subpopulation of tumor cell with self-renewal and tumorigenesis capabilities. CSCs are associated with cancer recurrence, progression, and chemoradiotherapy resistance. Establishing a reliable platform for CSC enrichment and study is a prerequisite for understanding the characteristics of CSCs and discovering CSC-related therapeutic strategies. Certain strategies for CSC enrichment have been used in laboratory, particularly fluorescence-activated cell sorting (FACS) and mammosphere culture. However, these methods fail to recapitulate the *in vivo* chemical and physical conditions in tumors, thus potentially decreasing the malignancy of CSCs in culture and yielding unreliable research results. Accumulating research suggests the promise of a biomaterial-based three-dimensional (3D) strategy for CSC enrichment and study. This strategy has an advantage over conventional methods in simulating the tumor microenvironment, thus providing a more effective and predictive model for CSC laboratory research. In this review, we first briefly discuss the conventional methods for CSC enrichment and study. We then summarize the latest advances and challenges in biomaterial-based 3D CSC platforms. Design strategies for materials, morphology, and chemical and physical cues are highlighted to provide direction for the future construction of platforms for CSC enrichment and study.

## Introduction

Cancer stem cells (CSCs) are a relatively rare subpopulation of tumor cells with self-renewal and tumorigenesis capabilities. These cells have been correlated with cancer recurrence, progression, and chemoradiotherapy resistance^1^. Understanding the characteristics of CSCs and discovering CSC-related drugs have important implications in anticancer therapy. A research prerequisite is the establishment of stable and repeatable culture conditions for the enrichment and study of CSCs while maintaining their stemness properties *in vitro*.

The conventional laboratory cell culture platform is two-dimensional (2D) and typically based on substrates optimized for the attachment of cells supplied with serum-rich medium, 21% oxygen and 5% CO_2_. However, CSCs are poorly adherent within tumors, under the conditions of restricted nutrients and oxygen^2^. Under typical 2D culture conditions, CSCs are forced to adhere and polarize on the rigid bottoms of dishes and are subjected to excessive nutrition and oxygen^3^; these conditions clearly fail to reflect the *in vivo* situation of CSCs in tumors. Inappropriate culture conditions may decrease the malignancy of CSCs^4^ and yield unreliable results in drug screening and mechanistic discovery investigations. To overcome the drawbacks of the 2D culture approach, three-dimensional (3D) culture models for CSC enrichment and study have been developed, among which fluorescence-activated cell sorting (FACS) and mammosphere culture models are most extensively used. Although these methods can establish 3D growth conditions for CSCs, they have limitations in their ability to represent the crosstalk between CSCs and environmental elements, such as the extracellular matrix (ECM), matrix stiffness, and other chemical and physical cues. Tumor slice culture, a 3D model originating decades ago, can retain the complexity of the original tumor microenvironment through culturing of tumor slices in selected conditions^5^. This model’s limitations include requirements for extensive cell manipulation and fresh tumor specimens, thus restricting its wide application.

In recent years, some biomaterial-based 3D strategies for CSC enrichment and study have been developed and provided advantages in simulating the tumor microenvironment *in vitro*. Organoids, a novel stem cell study model, have been introduced in the study of CSCs through the embedding of CSCs and other cancer-related cells in Matrigel or ECM-like biomaterials^6^. This model not only supports the CSC enrichment but also recapitulates the histopathology and cellular heterogeneity of tumors^7–10^. Additionally, unlike conventional study systems based on cancer cell lines, organoids can be generated from specimens of individual patients, thereby enabling the development of personalized therapeutic regimens^6^. The utilization of organoids in CSC studies has been reviewed by Drost et al.^6^. However, because technical difficulties exist in constructing organoids, and clinical specimens are usually needed, organoid studies are not achievable in every laboratory studying CSCs. For this reason, CSCs enriched from cancer cell lines remain the mainstream method used in CSC studies. Thus, in this review, we emphasize 3D platforms constructed for CSC enrichment from cancer cell lines.

Compared with CSCs obtained from conventional mammosphere culture, those grown on 3D scaffolds exhibit enhanced CSC characteristics^4,11^, thus suggesting that the biomaterial-based 3D culture platform can better maintain malignancy. Therefore, *in vitro* investigations performed on biomaterial-based 3D culture platforms may provide more effective and predictive data for clinical use. Here, we provide an overview of the conventional methods for CSC enrichment and study and summarize the latest advances and challenges in biomaterial-based 3D platforms for CSC enrichment and study, with an emphasis on the design of materials, morphology, and chemical and physical cues involved in the construction of the platforms.

## Conventional methods for CSC enrichment and study

**Figure 1:**
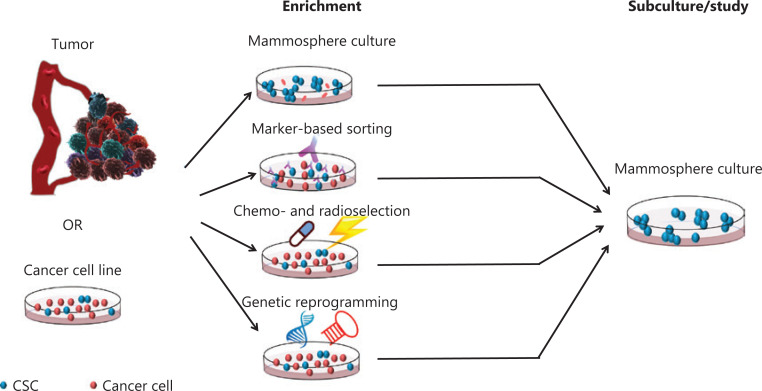
Schematic representation of conventional methods used for CSC enrichment and study. In laboratory settings, CSCs can be enriched from tumor or cancer cell lines. Mammosphere culture, also called suspension culture or spheroid culture, is the most extensively used method; this method aggregates CSCs spherically by culturing cells in suspension by using ultralow attachment plates, hanging drops, gyrator rotation and spinner flasks, or NASA rotary cell culture systems. Marker-based sorting is usually performed with FACS or MACS, according to the surface markers specifically expressed by CSCs. Chemo- and radioselection enriches CSCs by treating cells with anticancer drugs or radiation according to their chemo- and radioresistance characteristics. Genetic reprogramming is a novel method for CSC enrichment through epigenetic methods or genetic modifications. Regardless of the method through which CSCs are enriched, mammosphere culture is the common method for CSC subculture and study.

CSCs make up a minority of cells in heterogeneous tumors^1^. To study CSCs in laboratory settings, methods for the isolation, enrichment, and culture of CSCs are required. According to the characteristics of CSCs—such as their nonadherence, CSC-related gene expression, and chemo- and radioresistance—strategies for CSC enrichment have been developed, including mammosphere culture, CSC marker-based sorting, chemo- and radioselection, and genetic reprogramming (**Figure 1**). In these strategies, CSC marker-based sorting using fluorescence-activated cell sorting (FACS) and mammosphere culture are the most extensively used techniques for CSC isolation and enrichment. However, mammosphere culture is the common use method for the further study of CSCs, because CSCs isolated by FACS, chemo- and radioselection, or genetic reprogramming must be enriched by mammosphere culture, and mechanistic investigations and antidrug screening are based on mammospheres.

FACS selection is a strategy using flow cytometry to isolate CSCs according to the specific markers that they express. Since Bonnet et al.^12^ discovered CD34^+^CD38^-^ leukemia stem cells in the hematopoietic system in 1997, CSCs have been observed in various solid tumors exhibiting a tissue-specific antigenic phenotype. To date, several specific markers for CSCs have been identified, such as CD133 for breast, pancreas, prostate, brain and liver cancers; aldehyde dehydrogenase 1 (ALDH1) for breast, lung, ovarian, and colon cancer; CD44 for breast, pancreatic, colon, and prostate cancer; epithelial cell adhesion molecule (EpCAM) for stomach, pancreatic, and liver cancer; and CD166 for lung and colon cancer^12,13^. Through labeling of cells with fluorescent or magnetic bead-conjugated antibodies, the CSC population can be separated from tumors or cancer cells through FACS or magnetic-activated cell sorting (MACS). In addition to sorting through the use of surface markers, studies have also suggested a FACS approach according to the stem cell featured side population, through the use of Hoechst 33342^14,15^. In practice, FACS is preferred over MACS because its selection is purer and more accurate. However, FACS relies on costly dedicated equipment and consumes large amounts of antibodies, thus making FACS expensive and not accessible or affordable for every laboratory. Moreover, owing to cost concerns, FACS separation is usually performed on the basis of single biomarkers; however, no unique biomarker is acceptable for CSC identification^12^. Moreover, because of the effect of laser scanning in flow cytometry, the number of viable cells obtained by FACS is usually lower than the theoretical value.

In mammosphere culture, pioneered by Sutherland and colleagues in 1970^16^, and also called suspension culture or spheroid culture, cells are cultured in serum-free medium and kept in suspension through the use of an ultralow attachment plate, hanging drop, gyrator rotation and spinner flask, or NASA rotary cell culture system, which aggregates cells spherically and simulates nonadherent conditions *in vivo*^17^. Under suspension conditions, only CSCs, but not differentiated cells, such as cancer cells and stromal cells in tumors, survive and form floating colonies. Compared with FACS, mammosphere isolation of CSCs is technically more practical for laboratories lacking dedicated facilities and is feasible for the enrichment of multiple types of CSCs. However, interestingly, a recent study has suggested that mammosphere culture induces CSC enrichment in a cell line-dependent manner^18^.

In addition to FACS and mammosphere culture, some novel approaches for CSC enrichment have been elucidated. Because of the resistance of CSCs to chemotherapeutic agents and radiation, only CSCs are believed to be able to survive after treatment with chemotherapeutic drugs or radiotherapy. Studies have shown that cells expressing CSC surface markers are enriched after treatment with chemotherapeutic agents or radiation^19–21^, thereby suggesting a novel chemo- and radioselection approach for CSC enrichment.

According to the different expression profiles between CSCs and cancer cells, some studies have enriched CSCs by reprogramming cancer cells through epigenetic methods or genetic modification. Ikegaki et al.^22^ have reported a method of enriching stable neuroblastoma stem cells with tumor-initiating ability from neuroblastoma cells by treatment with epigenetic modifiers, DNA methylation (5AdC), and/or histone deacetylase (4-phenylbutyrate, or 4PB). Likewise, Mani et al.^23^ have generated CSCs by ectopically expressing factors involved in epithelial to mesenchymal transition (EMT) and have suggested that EMT is associated with the stem-like phenotype of cells^23^. However, these cells are genetically modified. Consequently, it will be interesting to directly compare these cells with those selected by FACS or mammosphere culture to ensure their reliability in preclinical research.

## Biomaterial-based CSC platforms

To maintain stemness *in vivo*, CSCs require the proper niche, including the ECM, cancer-associated fibroblasts, hypoxia, growth factors/cytokines, immunocytes, and other components^1^. In addition, biophysical cues in the tumor microenvironment, such as stiffness, porosity, topography, stretch, interstitial fluid flow, and compression, are crucial factors regulating the stem cell state of CSCs^24^. These chemical and physical elements maintain the malignant characteristics of CSCs, such as rapid proliferation, metastasis, and anticancer drug resistance.

Although mammosphere culture, the most commonly used conventional method to date, can mimic the *in vivo* nonadherent 3D growth morphology of CSCs to some extent by forming multicellular tumor spheroids *in vitro*, it cannot reestablish the interaction of CSCs with biochemical and biophysical cues in the niche. These drawbacks not only decrease the enrichment efficiency *in vitro* but also, more importantly, potentially provide less reliable data in the study of pathological mechanisms and drug screening^4^. Given the weaknesses of conventional methods, in recent years, some novel 3D CSC platforms based on natural and synthetic biomaterials have been developed for enrichment and study; these platforms have advantages in mimicking biochemical and biophysical elements in the tumor microenvironment and in allowing them to interact with cells.

### Biomaterials for platform construction

The ECM is the major component in the tumor niche, providing structural and biochemical support for the stemness phenotype of CSCs and regulating their fates^1,25^. The components of tumor ECM have been studied, mainly proteins, glycoproteins, proteoglycans, and polysaccharides^1,25^. According to these discoveries, biomaterials based on the natural components of the ECM or their analogs are generally used to construct platforms for CSC enrichment, thus not only providing 3D structural support for growth but also reestablishing the ECM-CSC interaction.

To simulate ECM-cell interaction, a decellularized amnion membrane scaffold containing the main components of the ECM has been proposed^26^. Likewise, commercially available Matrigel, a solubilized basement membrane matrix composed of certain ECM proteins, such as laminin and collagen IV, has been demonstrated to promote the enrichment of CSCs from adenocarcinoma cells, breast cancer cells, and prostate cancer cells^27^. Although these scaffolds have similar compositions to that of the ECM, potential variation may exist among scaffolds from different sources, and the exact components of scaffolds are usually unclear. To avoid potential variation and to generate a more controllable and repeatable research system, scaffolds with defined components are favored by researchers.

Hyaluronic acid (HA), a major glycosaminoglycan in the tumor ECM, has been demonstrated to be correlated with tumor angiogenesis, invasion, and metastasis^28^. However, although HA has good biodegradability and biocompatibility, its low mechanical strength and rapid bioabsorption rate have limited its use as the sole component in platform construction^29,30^. To exploit its advantages and bypass its disadvantages, HA is usually mixed with biopolymers with good mechanical properties, such as chitosan, to construct CSC platforms^29,30^ or is used as a modifier to improve enrichment efficiency, taking advantage of its ability to activate chemical signaling in cells^31^.

Collagens are the main structural element of the tumor ECM, and multiple collagen subtypes (e.g., collagen I, collagen III, and collagen IV) have been demonstrated to be associated with tumor initiation, EMT, drug resistance, and CSC self-renewal^25^. Platforms based on collagen I have been explored for the enrichment of liver CSCs^32^, colorectal CSCs^33^, and breast CSCs^34^.

Compared with costly natural ECM-based hydrogels or scaffolds, some naturally occurring polymers sharing similar molecular structures with tumor ECM components, such as alginate and chitosan, have attracted much attention^17,31,35–40^. Keratin, an inexpensive ECM-like protein with integrin-mediated cellular interactions, has been found to perform comparably to collagen I in facilitating tumor spheroid formation and CSC enrichment, thus suggesting that it is an economical material for CSC enrichment and study^33^. Alginate has a similar molecular structure to that of glycosaminoglycan in the ECM; is easily gelated in divalent cation solutions, such calcium chloride; and has good biocompatibility and low immunogenicity^41^. When used in cell culture, alginate is usually covalently modified by cell-binding ligands or peptides, such as RGD and cadherins, to facilitate cell adhesion, owing to its lack of a cell adhesion domain^41^. Rigid alginate hydrogels can be easily fabricated by modifying the concentration of alginate or calcium chloride^38,42^, thus providing a tool for exploring the effects of matrix stiffness on the biological behaviors of CSCs. Because of these merits, alginate hydrogels are extensively used in constructing platforms for CSC enrichment and study^17,31,35–40^.

Some synthetic materials are also used for CSC platforms, including poly(ε-caprolactone) (PCL)^43^, polydimethylsiloxane (PDMS)^44^, polyethylene glycol diacrylate (PEGDA)^45^, poly (allylamine hydrochloride) (PAH)^46^, and poly(2,4,6,8-tetravinyl-2,4,6,8-tetramethyl cyclotetrasiloxane) (pV4D4)^47^. Compared with natural biomaterials, synthetic materials enable experimental reproducibility by avoiding variations in the origin and purification of materials. Because they allow less chemical signaling, synthetic materials have advantages in investigating the influence of structural and physical cues by avoiding chemical crosstalk between cells and the chemical components of materials. Another merit of synthetic materials is that they are easier to functionalize with chemical groups, growth factors, drugs, and peptides, thereby enabling study of the role of chemical cues in a 3D microenvironment. Certain synthetic materials have been used in the study of cancer cells, such as polyethylene glycol (PEG), PDMS, PCL, poly(D,L-lactide-coglycolide) (PLG), and PLGA/polylactic acid (PLA)^48,49^, thus offering several candidates for the development of CSC platforms. However, whether these materials are qualified for enrichment and study of CSCs remains unclarified or controversial. For example, Palomeras et al.^43^ have demonstrated that PCL scaffolds improve the enrichment of breast CSCs, whereas Kievit et al.^40^ have reported that tumor spheroids cannot form on either PCL or chitosan-alginate scaffolds coated with PCL, thus suggesting that PCL is not suitable for the growth of glioblastoma CSCs.

Generally, on these platforms based on natural or synthetic materials, the proportion of cells expressing CSC-specific markers and the expression of stemness-related genes increases, and the chemo- and radioresistance of cells is improved over that of cells cultured on 2D substrate or 3D mammosphere culture conditions. This improvement is due to not only to the 3D structure constructed by materials but also to the chemical signaling provided by the chemical components of materials, as discussed in the following section. The reported natural and synthetic materials used to construct a 3D platform for CSC enrichment and study are summarized in **Table 1**.

**Table 1:** Reported biomaterial-based platforms for CSC enrichment and study

Material		Morphology	Cell line for enrichment	W/O cytokines	Reference
Natural material	Matrigel	Cell-laden disc	Adenocarcinomic human alveolar basal epithelial cells, A549 Human breast cancer cells, MCF7 Human prostate cancer cells, PC3	N	^27^
	HA + chitosan	Disc	Human glioblastoma multiforme, U118	N	^29^
	HA + chitosan	Disc	Human glioma, U87	N	^30^
	Collagen I + PEG	Cell-laden disc	Human hepatocyte cell line, HepG2	N	^32^
	Collagen I + keratin	Disc	Colorectal cancer cell line, HT29	Y	^33^
	Collagen I	Plate	Human breast cancer cells, MCF7	N	^34^
	Alginate	Microcapsule	Human cancer cell line, PANC-1	N	^36^
	Alginate	Microcapsule	Human pancreatic cell line, PC-3	Y	^17^
	Alginate	3D printed cell-laden plate	Human glioblastoma multiforme, U118	N	^35^
	Alginate	Cell-laden microbeads	Human hepatocellular carcinoma cells, HCCLM3 Human head and neck squamous cell carcinoma cells, TCA8113	N	^37,38^
	Alginate + HA	Disc	Mouse breast cancer cells, 4T1	Y	^31^
	Alginate + chitosan	Disc	Mouse prostate cancer cells, TRAMP-C2 Human breast cancer cells, MDA-MB-231 Human hepatocellular carcinoma, SK-Hep1	N	^39^
	Alginate + chitosan	Disc	Human glioblastoma cells, U87 Human glioblastoma multiforme, U118	N	^40^
	Fibrin gel	Cell-laden disc	Murine melanoma cells, B16-F1	N	^50^
Synthetic material	PCL	3D printed disc	Human breast cancer cells, MCF7	N	^43^
	PDMS	Disc	Human melanoma cells, WM115	N	^44^
	PEGDA	Cell-laden plate	Mouse breast cancer cells 4T1 Human breast cancer cells, MCF7	Y	^45^
	PAH/HA	Plate	Human hepatocellular carcinoma cells, HUH7	N	^46^
	pV4D4	Thin film	Human ovarian cancer cell line, SKOV3 Human breast cancer cell line, MCF-7 Human liver carcinoma cell line, Hep3B Human colon cancer cell line, SW480	N	^47^

### Morphology of biomaterial-based platforms

To construct a platform for CSC enrichment and study, materials are usually structured into discs/plates or microcapsules/microbeads. Through simple seeding of cells onto hydrogel or polymer thin films predeposited on cell culture dishes^31,33,34,47^ or sectioned presynthesized scaffolds^29,30,39,40^, 3D platforms for CSC culture and study can be constructed. However, in practice, the penetration depth of cell suspensions is limited by the surface properties of the material and the air pre-existing in the scaffolds, thus potentially resulting in the heterogeneous seeding of cells and the wasting of scaffolds. An alternative method is gelating cells with hydrogels to achieve a uniform distribution of cells^27,32,50^. In designing such cell-laden hydrogels, the pore size is a crucial issue that must be considered, because tumor spheroids usually grow into spheres larger than 100 µm in diameter, whereas hydrogels with smaller pore sizes may restrict the growth of CSCs. Additionally, nutrients may be adequately supplied for cells embedded in hydrogel^35^.

Alginate is an extensively explored biomaterial in constructing CSC platforms, because of its low cost and ECM-like properties. In the laboratory, alginate is usually mixed with cells, and cell-laden alginate microbeads are generated by extrusion of the mixture into calcium chloride solution with an electrostatic droplet generator or a syringe^37,38^. As discussed previously, pore size restriction and nutrient insufficiency may potentially affect the growth of CSCs. Rao et al.^17^ have developed a miniaturized 3D liquid core of microcapsules with an alginate hydrogel shell by using 2 syringes that push the core fluid with cancer cells and the shell fluid of sodium alginate. This microcapsule has been found to provide human prostate cancer cells with sufficient space and nutrients for the formation of tumor spheroids, and to allow the culture time to be shortened to 2 days while yielding CSC aggregates with better quality^17^. These findings have been supported by a direct comparison of microcapsules and microbeads by using embryonic stem cells, which has indicated a higher enrichment efficiency of microcapsules than microbeads. Sakai et al.^36^ have further improved the homogeneity of these microcapsules by templating the size and shape of cavities by using gelatin microparticles, which may improve the reproducibility of results obtained from the microcapsule platform. However, owing to the lack of physical attachment to the structural material, microcapsules do not support the investigation of cell-matrix interactions, such as the effect of matrix stiffness on the biological behaviors of CSCs.

A reproducible platform is a crucial premise for generating reproducible results in studies. The 3D bioprinting technique has advantages in constructing reproducible platforms, because it can precisely position biological materials, biochemicals, and living cells layer by layer, and enable spatial control of the placement of functional components^51^. Some 3D-bioprinted CSC platforms have been developed by using alginate and PCL^35,43^, and the effect of the scaffold angle on the enrichment of CSCs has been investigated^43^. However, the current platforms have not taken full advantage of 3D bioprinting techniques in generating 3D structures with well-defined geometries. For example, through precise design, an engineered 3D tumor microenvironment can be constructed by mimicking the *in vivo* distribution of tumor/nontumor cells, blood vessels, chemical components, and matrix rigidity. In this sense, 3D-bioprinted platforms have broad application potential in tumor studies by providing an engineered microenvironment combining various cellular and physicochemical factors. Additionally, because matrix topography has been demonstrated to determine the stem cell fate and EMT of cancer cells^52,53^, the inner structure of scaffolds or hydrogels can be designed with 3D bioprinting, which enables the study of the effect of topography in a 3D microenvironment.

**Figure 2:**
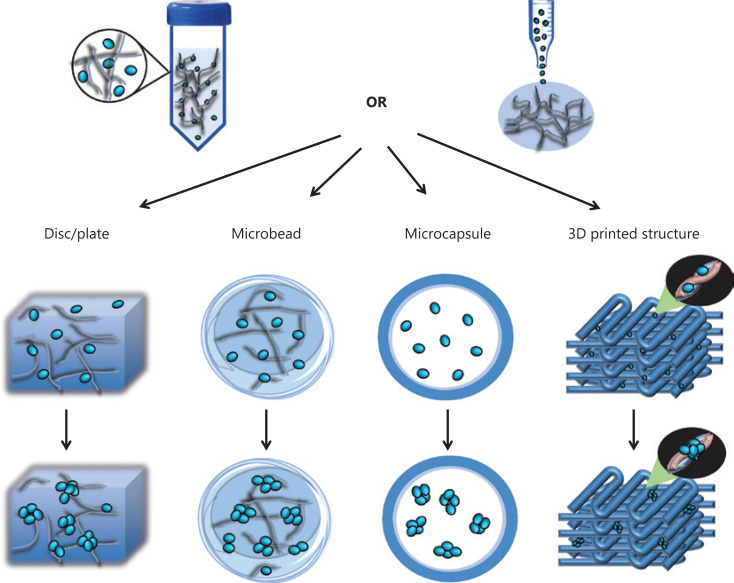
Schematic representation of the morphological design of the biomaterial-based platform for CSC enrichment and study. To construct CSC platforms, cell-laden hydrogels or presynthesized hydrogels/scaffolds are used to form discs/plates, microcapsules, microbeads, or defined 3D printed morphologies.

The morphology of biomaterial-based platforms for CSC enrichment and study is summarized in **Figure 2**.

### Chemical cues of biomaterial-based platforms

**Figure 3:**
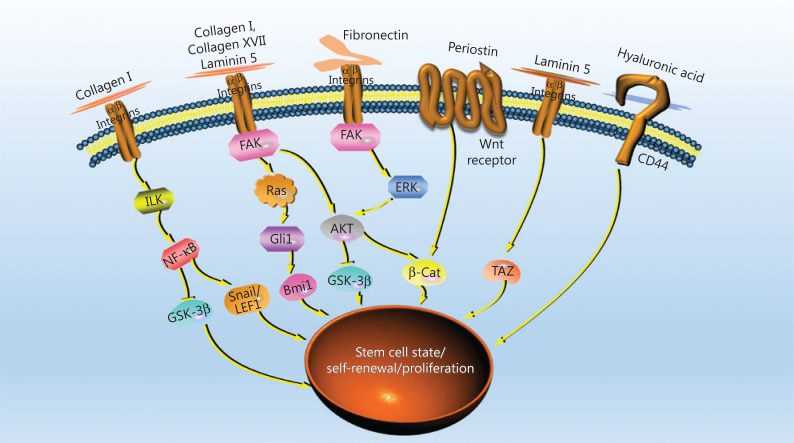
Signaling pathways correlating with the natural ECM components that regulate the stem cell state, self-renewal, and proliferation of CSCs. ILK: integrin-linked kinase; NF-κB: nuclear factor kappa B; GSK-3β: glycogen synthase kinase-3β; LEF1: lymphoid enhancer factor 1; FAK: focal adhesion kinase; AKT: also known as protein kinase B (PKB); ERK: extracellular signal-regulated kinase; β-cat: β-catenin; TAZ: transcriptional coactivator with PDZ-binding motif.

Biomaterial-based platforms are more than 3D structures supporting CSCs for growth; in fact, some biomaterials facilitate the enrichment of CSCs. On biomaterial-based platforms, CSC enrichment can be achieved without supplementation with the cytokines regularly used in conventional mammosphere culture (**Table 1**), such as epidermal growth factor (EGF), basic fibroblast growth factor (bFGF), N2, and B27, probably not only because of the 3D structural properties of the platform but also because of the chemical characteristics of materials in promoting CSC self-renewal, proliferation, and EMT^40^. The addition of regular cytokines in biomaterial-based culture further shortens the enrichment time^17^. Several natural ECM components have been demonstrated to be associated with the activation of signaling pathways regulating the stem cell state, self-renewal, and proliferation of CSCs^24,25,51,54^, thus indicating the merits of using natural ECM polymers in constructing CSC platforms. The signaling pathways correlated with the natural ECM polymers are summarized in **Figure 3**.

HA is one of the most notable biomaterials in CSC studies, because of its ability to bind the cellular receptor CD44, which activates downstream signaling pathways associated with cancer stemness, motility, and EMT^25,54,55^. Although the signaling molecule downstream of the HA-CD44 interaction has been poorly investigated in CSCs, studies nonetheless strongly suggest a role of HA in regulating CSC stemness and proliferation. Moreover, HA’s specific binding to CD44 further promotes the selection and enrichment of CSCs expressing CD44, such as liver, breast, prostate, ovarian, gastric, head, and neck CSCs^56^. Therefore, HA is an ideal candidate biomaterial for CSC enrichment or a modification material that can be combined with structural materials to improve the enrichment efficiency^31^. The advantage of HA in the enrichment of CD44-expressing CSCs suggests the possibility of using ligand-modified material in the exploitation of platforms, and accelerating CSC enrichment by targeting CSC-specific markers.

The structural material of the platform can be modified and functionalized with chemical groups, cytokines, growth factors, and peptides, depending on the culture requirements or study interests. Qiao et al.^31^ have immobilized EGF and bFGF—cytokines required for CSC enrichment in mammosphere culture—on alginate hydrogel, thus improving the enrichment efficiency of breast CSCs by prolonging the biological activities of these cytokines. Therefore, this method may provide a more economical culture strategy for CSCs. Likewise, some growth factors or cytokines, such as vascular endothelial growth factor (VEGF), a crucial factor in angiogenesis, can be engineered on scaffolds, thereby enabling study of angiogenesis in the tumor microenvironment. The immobilization of chemical reagents on materials, such as cytokines, growth factors, and proteases, might possibly simulate a microenvironment with cytokine or growth factor gradients in tumors.

Better hydrophilicity has been shown to facilitate the enrichment and stemness of prostate CSCs^57^, thus suggesting that the enrichment efficiency can be improved by modifying the surface properties of materials. This modification can be achieved through use of synthetic materials that can be easily functionalized with hydrophilic groups. Additionally, the covalent linking of the scaffold polymer to chemical groups or peptides enables the study of their functions in a 3D microenvironment. Synthetic materials have advantages in constructing engineered 3D platforms, owing to their characteristics of easy modification and low chemical crosstalk with cells.

### Physical cues of biomaterial-based platforms

Matrix stiffness in the tumor microenvironment is a major physical cue affecting CSCs. Tumors are stiffer than paracarcinoma tissues, because of the increased deposition of ECM components, whereas the stiffness distribution in tumors is heterogeneous^24^. Studies based on 2D substrates with determined rigidity have shown that matrix stiffness plays crucial roles in regulating the malignancy of cancer cells^58,59^. However, CSCs are low-adherent cells, and their connection to the adherent surface is unstable, thus possibly preventing CSCs from sufficiently sensing the physical properties of the substrate. Comparatively, 3D culture models allow for more physical contacts and interactions by embedding CSCs in hydrogels or scaffolds, which are superior to 2D substrates, particularly in the study of the response of CSCs to the physical microenvironment.

Alginate hydrogels are commonly used to construct 3D structures with different stiffnesses, ranging from hundreds to thousands of Pascals, by modulation of the concentration of alginate^31,38^ or the concentration of calcium chloride solution for cross-linking of alginate hydrogels^42^. Other natural or synthetic polymers, such as fibrin^50^ and polyethylene glycol diacrylate (PEGDA)^45^, have also been used to establish 3D structures with different stiffness. These models have revealed that an appropriate stiffness facilitates better enrichment and stemness of CSCs^31,38,45,50^, thus indicating that a more reliable and persuasive drug screening and mechanistic studies should be performed on hydrogels or scaffolds with appropriate stiffness according to the *in vivo* microenvironment.

However, to some extent, none of these biomaterials are ideal or satisfactory materials for fabricating different stiffnesses. First, as mentioned previously, chemical crosstalk exists between cells and the chemical components of biomaterials, particularly hydrogels or scaffolds constructed from natural ECM or ECM-like components. Hydrogels with different stiffnesses are usually generated by altering the concentrations of biomaterials^31,38,50^; however, in these research systems, the chemical effects of scaffold components cannot be excluded, because variations in concentration may result in different cell responses^31^. The chemical interaction between cells and material components could be avoided by the use of materials enabling fewer biochemical effects, such as synthetic materials. Second, together with changes in stiffness, other structural properties of scaffolds may also be altered, such as pore size, density, complexity, and roughness^38^, thus potentially affecting cell fate^60^. Therefore, owing to the chemical and structural variations in the construction of hydrogels or scaffolds with different stiffnesses, stiffness is not the sole factor responsible for the changes in cell behaviors. To minimize the concurrent adverse effects along with the change in stiffness, Liang et al.^32^ have developed a stiffness-controlled collagen-PEG gel and controlled the elastic modulus of the collagen hydrogel by incorporating varying amounts of PEG-diNHS into a pregel solution of collagen, thus allowing for the control of stiffness without significantly changing the permeability and number of cell adhesion motifs. By adjusting the concentration of Ca^2+^ in alginate, Lin et al.^42^ have fabricated hydrogels with different stiffnesses without-a significant change in inner structures.

The stiffness of the tumor microenvironment is one of the major physical cues affecting CSC fate, and therefore has been a major focus in recent 3D biomaterial-based studies. Instead of biomaterial platforms with uniform mechanical properties, a recent study has presented a system constructed with alginate and near-infrared (NIR) light-triggered liposomes, which allows for both spatial and temporal control of stiffness by light^61^. Because mechanical heterogeneity of the ECM exists in tumors^62^, this system better mimics the mechanical gradient in tumors.

In addition to matrix rigidity, other physical factors control the biological behaviors of CSCs, such as shear stress in blood vessels and interstitial fluid, compression, and matrix topography^24^. To date, the influences of these physical factors on CSCs have been investigated in only 2D conditions; 3D engineered models enabling the study of these physical factors have rarely been reported.

## Future prospects

**Figure 4:**
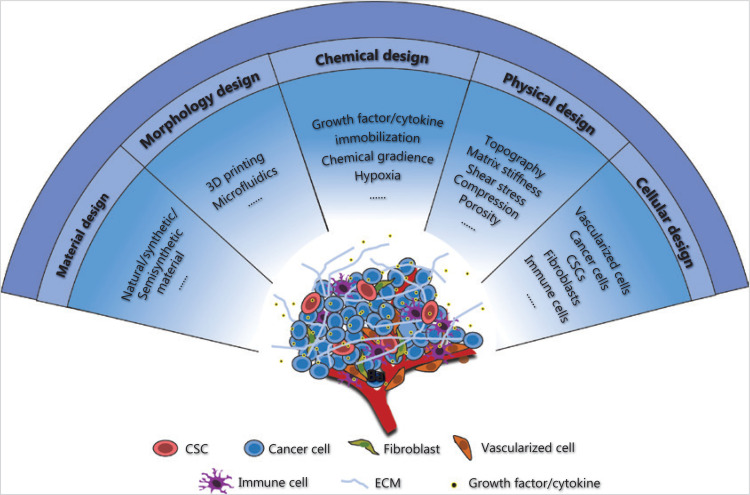
Schematic representation of the composite biomaterial-based platform for CSC enrichment and study. To better mimic the *in vivo* microenvironment of tumors and obtain valid, reliable, and credible research data, prospective CSC studies should be based on a composite platform, considering multiple cellular and chemicophysical elements.

CSCs are a rare tumor cell subpopulation responsible for tumor progression, metastasis, drug resistance, and recurrence. The *in vivo* tumor microenvironment is complex, comprising various cellular and chemicophysical elements. To obtain reliable and credible laboratory data, and to bridge the gap from laboratory studies to clinical use, a CSC enrichment and study platform should be constructed by simulating the tumor microenvironment to the greatest extent possible. On the basis of the understanding of the tumor microenvironment, and the development of biomaterials and their related fabrication techniques, composite platforms could be constructed by considering multiple factors, including blood vessels, hypoxia, cancer/noncancer cells, growth factors/cytokines, and mechanical cues (**Figure 4**).

The construction of a CSC platform that better mimics the characteristics of these elements in tumors requires the development of biomaterial technology, which could potentially enable the establishment of spatially and temporally designed distributions of biomechanical and biochemical elements in a 3D platform^61^ and allow for vascularization^63^. Moreover, with techniques for measuring and simulating cell–ECM interactions, the interactions between CSCs and the surrounding matrices could be investigated at the cellular and subcellular levels, over biologically relevant time scales^64,65^. The methodological improvement of biomaterials would facilitate better mimicry of the tumor microenvironment in the laboratory and greatly improve the outcomes of drug screening and the understanding of tumor development and heterogeneity.
